# Contribution for new genetic markers of rheumatoid arthritis activity and severity: sequencing of the tumor necrosis factor-alpha gene promoter

**DOI:** 10.1186/ar2173

**Published:** 2007-04-04

**Authors:** João Eurico Fonseca, João Cavaleiro, José Teles, Elsa Sousa, Valeska L Andreozzi, Marília Antunes, Maria A Amaral-Turkman, Helena Canhão, Ana F Mourão, Joana Lopes, Joana Caetano-Lopes, Pamela Weinmann, Marta Sobral, Patrícia Nero, Maria J Saavedra, Armando Malcata, Margarida Cruz, Rui Melo, Araceli Braña, Luis Miranda, José V Patto, Anabela Barcelos, José Canas da Silva, Luís M Santos, Guilherme Figueiredo, Mário Rodrigues, Herberto Jesus, Alberto Quintal, Teresa Carvalho, José A Pereira da Silva, Jaime Branco, Mário Viana Queiroz

**Affiliations:** 1 Rheumatology Research Unit, Instituto de Medicina Molecular, Faculdade de Medicina, Universidade de Lisboa, Av. Professor Egas Moniz, 1649-028, Lisboa, Portugal; 2 Santa Maria Hospital, Av. Professor Egas Moniz, 1649-035, Lisboa, Portugal; 3 Escola Nacional de Saúde Pública Sérgio Arouca, R. Leopoldo Bulhões, 1480, 21031-210, Rio de Janeiro, Brasil; 4 Centro de Estatística e Aplicações, Faculdade de Ciências, Universidade de Lisboa, Campo Grande, 1749-016, Lisboa, Portugal; 5 Egas Moniz Hospital, Rua da Junqueira, 126, 1349-019, Lisboa, Portugal; 6 Coimbra University Hospital, Praceta Mota Pinto, 3000-075, Coimbra, Portugal; 7 Faro Hospital, Rua Leão Penedo, 8000-386, Faro, Portugal; 8 Nossa Senhora da Assunção Hospital, Rua D. Alexandrina Soares de Albergaria, 6270-498, Seia, Portugal; 9 Caldas da Rainha Hospital, Largo Rainha Dona Leonor, 2500-176, Caldas da Rainha, Portugal; 10 Portuguese Institute of Rheumatology, Rua da Beneficência, 7, 1050-034, Lisboa, Portugal; 11 Infante D. Pedro Hospital, Avenida Artur Ravara, 3814-501, Aveiro, Portugal; 12 Garcia de Orta Hospital, Av. Torrado da Silva, 2801-951, Almada, Portugal; 13 Divino Espírito Santo Hospital, Praça 5 de Outubro, 9500, Ponta Delgada, Portugal; 14 Funchal Central Hospital, Avenida Luís de Camões, 9000, Funchal, Portugal; 15 Cell Biology Unit, Instituto de Medicina Molecular, Faculdade de Medicina, Universidade de Lisboa, Av. Professor Egas Moniz, 1649-028, Lisboa, Portugal

## Abstract

The objective of this study was to assess whether clinical measures of rheumatoid arthritis activity and severity were influenced by tumor necrosis factor-alpha (*TNF-α*) promoter genotype/haplotype markers. Each patient's disease activity was assessed by the disease activity score using 28 joint counts (DAS28) and functional capacity by the Health Assessment Questionnaire (HAQ) score. Systemic manifestations, radiological damage evaluated by the Sharp/van der Heijde (SvdH) score, disease-modifying anti-rheumatic drug use, joint surgeries, and work disability were also assessed. The promoter region of the *TNF-α *gene, between nucleotides -1,318 and +49, was sequenced using an automated platform. Five hundred fifty-four patients were evaluated and genotyped for 10 single-nucleotide polymorphism (SNP) markers, but 5 of these markers were excluded due to failure to fall within Hardy-Weinberg equilibrium or to monomorphism. Patients with more than 10 years of disease duration (DD) presented significant associations between the -857 SNP and systemic manifestations, as well as joint surgeries. Associations were also found between the -308 SNP and work disability in patients with more than 2 years of DD and radiological damage in patients with less than 10 years of DD. A borderline effect was found between the -238 SNP and HAQ score and radiological damage in patients with 2 to 10 years of DD. An association was also found between haplotypes and the SvdH score for those with more than 10 years of DD. An association was found between some *TNF-α *promoter SNPs and systemic manifestations, radiological progression, HAQ score, work disability, and joint surgeries, particularly in some classes of DD and between haplotypes and radiological progression for those with more than 10 years of DD.

## Introduction

Tumor necrosis factor-alpha (TNF-α) has been shown to be relevant for the physiopathology of rheumatoid arthritis (RA), and its inhibition by anti-TNF-α antibodies or recombinant soluble receptors results in a major improvement of this disease [1]. On the other hand, TNF-α production shows a wide variation, with high- and low-producer phenotypes present in humans [2] but with a strong concordance in monozygotic twins [3], pointing to the influence of genetic variation on the regulation of TNF-α circulating levels. These arguments have favored the view that genetic factors controlling TNF-α could have a major impact on RA outcome. An extensive network of gene products is involved in the production, modulation, and decay of TNF-α, affecting the stabilization of the transcripts, full activation of membrane-bound TNF-α by proteases, and the interaction with its membrane receptors and with membrane-shedded receptors [4]. In addition, the gene itself and/or its promoter area could be the source of genetic variation. However, present knowledge suggests that the highest genetic variability is concentrated in the promoter area of the *TNF-α *gene, where at least eight different single-nucleotide polymorphisms (SNPs) are concentrated, with the potential to affect the binding of transcriptor factors and thus to control the activity of the promoter and resulting mRNA and protein levels [4].

Several studies have addressed the issue of *TNF-α *gene promoter SNPs and RA outcome. Although some contradictory results have emerged, the data published so far indicate the possible existence of *TNF-α *gene promoter variants that act as markers for disease severity and response to treatment in RA [5-7]. Nevertheless, further investigation is necessary to determine whether the previously identified *TNF-α *gene promoter variants contribute directly to RA outcome or act as genetic markers of other polymorphisms in the *TNF-α *gene promoter area.

In this study, we have analyzed the promoter region of the *TNF-α *gene, between nucleotides -1,318 and +49, of 554 patients with RA from 11 Portuguese rheumatology centers serving the mainland and Azores and Madeira Islands. An association was found between some SNPs, localized in the -238, -308, -857, and -863 positions, and systemic manifestations, functional status, radiological damage, work disability, and joint surgeries and between haplotypes and radiological damage for those with more than 10 years of disease duration (DD).

## Materials and methods

### Patients

Patients included in this study (*n *= 554) fulfilled the American College of Rheumatology (ACR) 1987 revised criteria for RA [8]. Research was carried out in compliance with the Declaration of Helsinki. Written informed consent was obtained from all patients, and all of the ethics committees of the participating hospitals approved the study. Patients were randomly selected and evaluated at Santa Maria Hospital (Lisbon), Egas Moniz Hospital (Lisbon), Coimbra University Hospital (Coimbra), Faro Hospital (Faro), Nossa Senhora da Assunção Hospital (Seia), Caldas da Rainha Hospital (Caldas da Rainha), the Portuguese Institute of Rheumatology (Lisbon), Infante D. Pedro Hospital (Aveiro), Garcia de Orta Hospital (Almada), Divino Espírito Santo Hospital (Ponta Delgada), and Funchal Central Hospital (Funchal). For every patient included in this study, detailed data were collected in a separate clinical record [9]: DD, age of onset, rheumatoid factor (RF), erythrocyte sedimentation rate (ESR) and C-reactive protein at the time of evaluation, the number of previous disease-modifying anti-rheumatic drugs (DMARDs), the use of anti-TNF-α treatments, the dose of prednisolone, previous joint surgeries due to inflammatory destructive arthropathy directly related to RA (total joint replacement and arthrodesis), the number of years of education, and work disability (defined as the legal incapacity to work as judged by an official medical committee and directly attributed to the consequences of RA). Patients were considered to have systemic manifestations if at least one of the following clinical features could be detected: subcutaneous nodules, pulmonary fibrosis confirmed by chest roentgenograms and lung function tests, echocardiographic evidence of pericardial effusion, pleural effusion shown by chest roentgenograms, Felty syndrome (less than 2 × 10^9^/l granulocytes and splenomegaly), cutaneous vasculitis (leukocytoclastic vasculitis histologically proved), non-compressive neuropathy confirmed by electromyography, or the diagnosis of Sjögren syndrome based on the clinical symptoms of dry eyes and dry mouth (confirmed by a positive Schirmer's test and/or keratoconjunctivitis sicca with involvement of salivary glands documented by positive lip biopsy and/or salivary scintigraphy). Simple x-rays of the hands and feet were analyzed with the Sharp/van der Heijde (SvdH) method [10]. Disease activity was evaluated according to the core disease activity parameters proposed by the ACR and the European League Against Rheumatism: number of swollen and tender joints, pain as evaluated by the patient in a 10-cm analogue scale, disease activity as evaluated by the patient and the physician in a 10-cm analogue scale, ESR, and Health Assessment Questionnaire (HAQ) score [11]. The disease activity score using 28 joint counts (DAS28) [12] was calculated. The presence of other RA cases in the family was recorded if confirmed by a rheumatologist.

### Genotyping for polymorphisms

Genomic DNA was extracted from heparin anticoagulated whole blood, after sedimentation at room temperature, using the commercial kit QIAamp^® ^DNA blood mini kit (QIAGEN Inc., Valencia, CA, USA) according to the manufacturer's instructions. For the *TNF-α *promoter nucleotide sequencing, a 1,367-base pair fragment of the *TNF-α *5' flanking region, spanning from position -1,318 to +49, was amplified by polymerase chain reaction (PCR), using the forward primer F1: 5'-GAAAGCCAGCTGCCGACCAG-3' and the reverse primer R1: 5'-CCCTCTTAGCTGGTCCTCTGC-3', designed according to human *TNF-α *sequence (gi:27802684). PCRs were performed in a 50-μl reaction volume, using 10 μM of each primer, 5 μl of 10× reaction buffer [160 mM (NH_4_)_2_SO_4_, 670 mM Tris-HCl (pH 8.8), 0.1% Tween-20] (Bioline Ltd., London, UK), 1.5 mM MgCl_2_, 0.2 mM dNTPs, and 1 U of BioTaq polymerase (Bioline Ltd.). A 100-ng aliquot of genomic DNA was denatured for 5 minutes at 94°C followed by 35 cycles of amplification (45 seconds at 95°C, 45 seconds at 66°C, and 45 seconds at 72°C) and a final extension step at 72°C for 10 minutes. PCR products were subsequently purified trough incubation with 1 U of ExoSAP-IT (Amersham, now part of GE Healthcare, Little Chalfont, Buckinghamshire, UK) at 37°C for 15 minutes and then at 80°C for an additional 15 minutes. All of the thermal reactions took place in 96-well microtiter plates on a T1 Thermocycler (Biometra, Goettingen, Germany).

DNA direct sequence analysis was carried out with a BigDye^® ^Terminator version 3.1 Cycle Sequencing Kit (Applied Biosystems, Foster City, CA, USA) according to the manufacturer's instructions. A 300-ng aliquot of DNA template and the internal overlap primers F2: 5'-TGTGACCACAGCAATGGGTAGG-3', F3: 5'-CCAAACACAGGCCTCAGGACTC-3', and R5: 5'-GAAAGCTGAGTCCTTGAGGGAG-3' were used. Sequencing reactions were carried out with an initial step of 1 minute at 96°C, followed by one cycle of 96°C for 10 seconds and 60°C for 4 minutes (25 times). Purification of sequencing reactions was performed by ethanol precipitation. The purified reaction mixture was analyzed on a four-capillary automated sequencer ABI PRISMR 3100-Avant Genetic Analyzer (Applied Biosystems) with Sequencing Analysis Software version 5.2 (Applied Biosystems).

### Statistical analysis

Multiple linear regression models were used to assess the association between the five polymorphisms and DAS28 and HAQ score. For systemic manifestations, work disability, joint surgeries, and RF, logistic regression models were used instead. All the analyses were adjusted for the effects of socio-demographic and clinical covariates. Except for RF, the models were stratified by DD (less than 2 years, 2 to 10 years, and more than 10 years). In regression models, SNPs were separated into two groups: more prevalent homozygous versus heterozygous (aggregated with the less prevalent homozygous).

**Figure 1:**
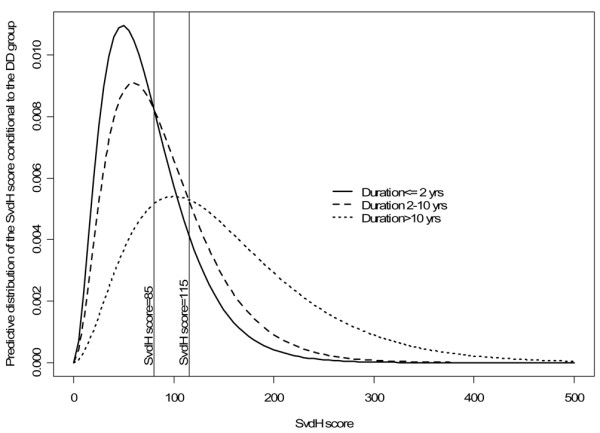
Estimated distribution of the Sharp/van der Heijde (SvdH) score according to the duration of the disease (DD).

**Figure 2:**
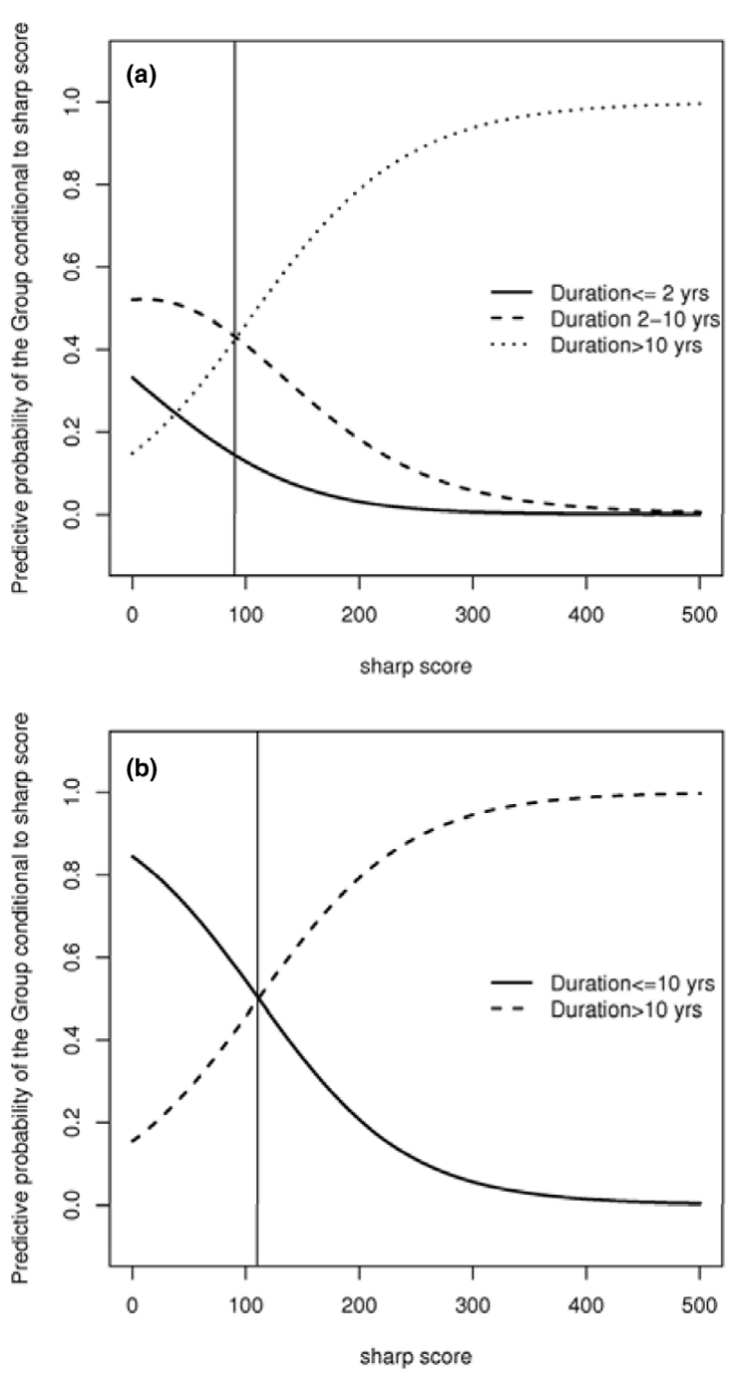
'Competition' allocation curves. **(a) **Predictive probability of patients with more than 10 years of disease duration (DD), those with 2 to 10 years of DD, and those with less than 10 years of DD as a function of the Sharp/van der Heijde (SvdH) score. **(b) **Predictive probability of patients with more than 10 years of DD and those with less than 10 years of DD as a function of the SvdH score.

SvdH score is known to be strongly associated with DD. To estimate the distribution of the SvdH score in the three different age groups, a hierarchical Bayesian model was built [13]. For each group, the SvdH score was considered to follow a gamma distribution with a shape parameter equal to 3 and scale parameter β_i_, i = 1, 2, 3, with i representing the groups. These β_i _values were supposed to be independent *a priori *with a non-informative distribution. The group distribution was considered to be categorical with probabilities π_1_, π_2_, and π_3 _following *a prior *Dirichlet distribution. These estimated probability curves (predictive conditional densities of the score) for the scores are essentially descriptive and should not be used for classification purposes. These curves are expected, one at a time as the SvdH score value increases, to exhibit higher values than the other two curves. The points at which the curves intersect can be seen as cutoff points. As can be seen in Figure 1, two cutoff points are expected to be found. The cutoff points found are 85 and 115, meaning that SvdH score values below 85 are more likely to occur in the first group, values between 85 and 115 are more likely to occur in the second group, and values above 115 are more likely to occur in the third group. Conversely, for classification purposes, the SvdH score of a particular patient can be used to calculate the conditional probability of his or her belonging to a given group and, once this has been done for each group, to classify the patient in the group for which the conditional probability is highest. These calculations take into account the way the patients distribute along the groups, namely the weight of each group in the sample. The predictive probability of the group as a function of the SvdH score (Figure 2a) showed that patients with less than 2 years and those with 2 to 10 years of DD could not be separated into two groups by any SvdH score cutoff point. The method classifies them all as belonging to the second group. However, an SvdH score close to 90 points could distinguish two groups: patients with less than 10 years and those with more than 10 years of DD. The patients were then separated into these two groups and distributions were recalculated. The value of 110 points was found to be a sensible choice for the cutoff point for the SvdH score (Figure 2b). This cutoff point was confirmed using the cross-validation procedure by dividing the available dataset into an experimental group (2/3 of the data) and a test group (1/3). This approach allowed us to classify the patient as having a 'good prognosis,' as having an 'expected evolution of the disease,' or as having a 'bad prognosis.' Table 1 shows that this cutoff point classified 67.4% of the patients as having had a disease course as theoretically expected (long DD and high SvdH score or short DD and low SvdH score), 20.6% as having had a disease course milder than theoretically expected, possibly revealing a subset of patients with good prognosis (long DD and low SvdH score), and 12.0% as having had a disease course worse than theoretically expected, possibly depicting a subset of patients with bad prognosis (short DD and high SvdH score). Generalized linear models with gamma distribution and logarithmic link function were used to study the association between genotypes and SvdH score, also adjusted for the effects of socio-demographic and clinical covariates.

**Table 1:** Association of Sharp/van der Heijde score and disease duration

	Disease duration	
	
Sharp/van der Heijde score	≤10 years	>10 years
≤110	121 (38.3%)	65 (20.6%)
>110	38 (12.0%)	92 (29.1%)

*P *value of χ^2 ^test is less than 0.001.

All SNP analyses, including conformance with Hardy-Weinberg equilibrium, linkage disequilibrium, and haplotype frequency estimation, were performed using Haploview version 3.32 [14]. Conformance with Hardy-Weinberg equilibrium was computed using an exact test [14], pairwise standardized disequilibrium coefficient measures (D') were calculated between each marker, and haplotype frequencies were estimated using an accelerated expectation-maximization (EM) algorithm. Association of haplotypes with covariates was assessed by χ^2 ^test. The public domain software R (2006, R Development Core Team) [15] was used to process the regression analysis.

## Results

### Patient characteristics

The 491 patients who were effectively included in the analysis had a mean age of 57 ± 13.3 years, 85% were women, mean DD was 12.7 ± 10.5 years, RF was detected in the serum of 319 patients (65%), systemic manifestations were present in 124 patients (25%), the mean DAS28 was 4.1 ± 1.5, the mean HAQ score was 1.2 ± 0.8, the mean modified SvdH score was 117.2 ± 77.8, 113 patients (23%) had been submitted to joint surgeries, and 282 (57%) were not working due to RA. The patients had been previously treated with 2.3 ± 1.6 DMARDs. The characteristics of the RA population studied were similar to the disease pattern previously described in Portugal [16] (Table 2).

**Table 2:** Patient characteristics

		Disease duration			
Characteristics	Total	<2 years	2–10 years	>10 years	Percentage of data missing

Number of patients (percentage)	491	70 (14)	174 (36)	236 (48)	-
Females/males, *n *(percentage)	417/74 (85/15)	50/10	150/24	206/30	-
Age at disease onset in years, mean (SD)	57.4 (13.3)	55.4 (16.8)	54.8 (14.0)	60.0 (11.0)	2%
Years of education, mean (SD)	5.8 (4.3)	6.0 (4.5)	7.0 (4.7)	4.8 (3.7)	7%
Family history of RA, *n *(percentage)^a^	67/400 (14/81)	8/62	18/152	41/183	5%
Disease duration in years, mean (SD)	12.7 (10.5)	-	-	-	2%
Number of previous DMARDs, mean (SD)	2.3 (1.6)	1.3 (1.1)	2.1 (1.4)	2.7 (1.8)	2%
Dose of prednisolone in milligrams, mean (SD)	5.9 (3.7)	6.1 (4.1)	5.8 (3.8)	5.8 (3.4)	3%
Biologics, *n *(percentage)^a^	150/339 (30/69)	4/65	53/121	150/85	1%
Health Assessment Questionnaire score, mean (SD)	1.18 (0.8)	0.97 (0.7)	1.04 (0.8)	1.36 (0.9)	6%
DAS28, mean (SD)	4.14 (1.5)	4.4 (1.7)	4.1 (1.5)	4.0 (1.5)	3%
Submitted to joint surgery, *n *(percentage)^a^	113/366 (23/74)	1/68	21/152	91/143	3%
Work disability, *n *(percentage)^a^	282/200 (57/41)	38/32	80/84	161/74	2%
Systemic manifestations, *n *(percentage)^a^	124/357 (25/73)	8/62	32/141	81/154	2%
Rheumatoid factor, *n *(percentage)^a^	319/124 (65/25)	44/22	106/52	166/48	10%
Sharp/van der Heijde score, mean (SD)	117.2 (77.8)	-	-	-	34%

^a^Values presented indicate yes/no. DAS28, disease activity score using 28 joint counts; DMARD, disease-modifying anti-rheumatic drug; RA, rheumatoid arthritis; SD, standard deviation.

### Effect of genetic variants on disease activity and outcome measures

A total of 554 patients were evaluated and genotyped for 10 SNP markers, and five of these markers were excluded due to failure to fall within Hardy-Weinberg equilibrium or to monomorphism. For the remaining five markers (positions -1,036, -863, -857, -308, and -238), haplotypes were constructed using an accelerated EM algorithm implemented in the Haploview software. The subsequent analysis was carried out in a subgroup of 491 Caucasian patients with at least one of the five SNPs identified. In the analysis of the five SNPs (Table 3), patients with more than 10 years of DD presented significant associations between the -857 SNP and systemic manifestations (*p *< 0.05), as well as joint surgeries (*p *< 0.01). Associations were also found between the -308 SNP and work disability in patients with 2 to 10 years (*p *< 0.05) and those with more than 10 years (*p *< 0.01) of DD. DAS28 was weakly associated (*p *< 0.10) with the -863 SNP for those with a DD of less than 2 years and with the -308 SNP for patients with from 2 to 10 years of DD.

**Table 3:** Association of tumor necrosis factor-alpha gene promoter polymorphisms with rheumatoid arthritis activity and outcome measures

		DAS28^a^		HAQ		Systemic manifestations		Work disability		Joint surgery		RF^b^		SvdH score	
		
		Coeff	*P *value	Coeff	*P *value	Coeff	*P *value	Coeff	*P *value	Coeff	*P *value	Coeff	*P *value	Coeff	*P *value
<2 years	1036TT	1.111	0.280	0.367	0.505	-	-	-	-	-	-				
	863CC	-1.911	0.081	-0.799	0.178	-	-	-	-	-	-				
	857CC	-1.150	0.116	-0.200	0.614	-	-	-	-	-	-				
	308GG	0.154	0.827	0.357	0.480	-	-	-	-	-	-				
	238GG	-0.646	0.507	-0.878	0.148	-	-	-	-	-	-				

2 to 10 years	1036TT	-0.071	0.912	-0.152	0.639	-0.482	0.690	-0.946	0.351	0.965	0.480				
	863CC	-0.286	0.661	0.147	0.659	0.050	0.970	0.686	0.514	-1.992	0.160				
	857CC	-0.289	0.314	0.074	0.620	-0.384	0.480	-0.773	0.093	-0.829	0.190				
	308GG	-0.507	0.080	0.133	0.395	-0.380	0.510	-1.133	0.027	0.255	0.770				
	238GG	-0.106	0.875	0.670	0.052	0.497	0.710	-0.917	0.415	-0.598	0.680				

>10 years	1036TT	-0.023	0.966	-0.450	0.187	-0.704	0.417	-0.642	0.458	-1.481	0.133				
	863CC	-0.037	0.944	0.548	0.099	0.359	0.663	1.253	0.136	1.512	0.117				
	857CC	-0.002	0.996	0.036	0.834	-0.860	0.049	0.076	0.873	-1.217	0.006				
	308GG	-0.151	0.563	0.041	0.800	-0.337	0.427	1.736	0.002	0.496	0.257				
	238GG	-0.029	0.953	0.268	0.378	-1.308	0.099	0.525	0.522	1.088	0.253				

	1036TT											-0.422	0.512		
	863CC											1.142	0.072		
	857CC											0.559	0.079		
	308GG											0.015	0.964		
	238GG											1.089	0.062		

<10 years	1036TT													-0.103	0.676
	863CC													-0.062	0.809
	857CC													-0.110	0.375
	308GG													-0.299	0.039
	238GG													-0.315	0.255

>10 years	1036TT													-0.003	0.992
	863CC													0.222	0.405
	857CC													-0.129	0.320
	308GG													0.041	0.731
	238GG													0.481	0.070

Coefficients were adjusted for gender, education, disease-modifying anti-rheumatic drugs, prednisolone dose, use of biologics, family history, and hemoglobin. ^a^Model was not adjusted for hemoglobin; ^b^analysis was not stratified for disease duration. Coeff, coefficients; DAS28, disease activity score using 28 joint counts; HAQ, Health Assessment Questionnaire; RF, rheumatoid factor; SvdH, Sharp/van der Heijde.

A borderline effect was found between the -238 SNP and HAQ score in patients with 2 to 10 years of DD (*p *= 0.052), and a weak association (*p *< 0.10) was detected between the -863, -857, and -238 SNPs and RF. Due to a lack of data in some of the covariates, the models for systemic manifestations, work disability, and joint surgery applied to the group of patients with less than 2 years of DD were not able to make an accurate estimation of the association (Table 3).

Using the previously described model for x-ray analysis, we found an association between the -308 SNP and SvdH score in patients with less than 10 years of DD (*p *< 0.05). For those with more than 10 years of DD, a weak association between the -238 SNP and SvdH score was detected (*p *< 0.10). Given that combinations of allelic variants at each of the five markers may provide valuable information regarding functional variation in terms of TNF-α transcription, we estimated haplotype frequencies for each particular subgroup of patients and evaluated its association with clinical covariates in addition to analyzing the association of individual SNPs with clinical covariates (Table 4). This analysis revealed an association between haplotypes and the SvdH score for those with more than 10 years of DD (Table 4).

**Table 4:** Association of tumor necrosis factor-alpha gene promoter haplotypes with rheumatoid arthritis activity and outcome measures

	HAQ			DAS28				Systemic manifestation			SvdH score		
		<1	>1		<2.6	2.6–5.1	>5.1		No	Yes			
	
<2 years	CACAG	1	0	CACGG	3	6	5	CACAG	1	0			
	CACGG	7	8	CCCGA	1	1	0	CACGG	13	1			
	CCCGA	1	2	TCCAG	1	1	1	CCCGA	2	0			
	TCCAG	1	1	TCCGG	9	17	14	TCCAG	2	1			
	TCCGG	25	17	TCTGG	0	2	1	TCCGG	38	5			
	TCTGG	2	1					TCTGG	2	2			
	
	*P *value	0.80		*P *value	0.95			*P *value	0.24				

		<1	>1		<2.6	2.6–5.1	>5.1		No	Yes			
	
2 to 10 years	CACGG	12	14	CACGG	5	15	6	CACGG	21	5			
	CCCGA	6	2	CCCGA	2	3	3	CCCGA	7	1			
	TCCAG	11	7	CCCGG	1	1	1	TCCAG	16	2			
	TCCGG	48	41	TCCAG	1	11	2	TCCGG	74	17			
	TCTGG	12	7	TCCGG	18	50	25	TCTGG	17	3			
				TCTGG	2	12	6						
	
	*P *value	0.57		*P *value	0.81			*P *value	0.93				

		<1	>1		<2.6	2.6–5.1	>5.1		No	Yes		<110	>110
	
>10 years	CACGG	21	31	CACGG	13	27	14	CACGG	37	16	CACGG	3	4
	CCCGA	2	6	CCCGA	1	4	2	CCCGA	3	6	CCCGA	6	1
	TCCAG	13	16	TCCAG	5	18	8	TCCAG	20	11	TCCAG	10	2
	TCCGG	48	61	TCCGG	18	67	29	TCCGG	79	38	TCCGG	5	8
	TCTGG	7	8	TCTGG	3	10	4	TCTGG	10	6	TCTGG	9	7
	
	*P *value	0.85		*P *value	0.98			*P *value	0.30		*P *value	0.09	

												<110	>110
	
<10 years											CACGG	3	9
											CCCGA	7	4
											TCCAG	10	5
											TCCGG	8	2
											TCTGG	1	6
	
											*P *value	0.01	

DAS28, disease activity score using 28 joint counts; HAQ, Health Assessment Questionnaire; SvdH, Sharp/van der Heijde.

## Discussion

An association was found between some SNPs (-857, -308, and -238) and systemic manifestations, radiological progression, work disability, and joint surgeries. A weak association was also observed between other SNPs (-863 and -238) and DAS28, HAQ score, and RF, particularly in some categories of DD. In addition, an association between haplotypes and radiological progression for those with more than 10 years of DD was detected.

Previous reports regarding the -308 position are contradictory, and different works suggest that both the -308GG [17] and -308GA [18] genotypes are implicated in increased RA severity. In line with this controversy, several independent groups using reporter gene constructs have shown that the -308A allelic variation presents a higher transcriptional activity than the -308G form [19-21], although other studies were unable to show any difference between the transcriptional activity of -308G and -308A alleles [22,23]. Nevertheless, more recent data suggest a direct functional effect of the -308 SNP by modifying the binding of transcription factors [24]. On the other hand, the -857CC genotype appears to be associated with a worse response to etanercept in patients with RA [25], and studies in healthy controls have shown higher production of TNF-α in whole-blood cultures stimulated with lipopolysaccharide in individuals homozygous for the -857C allele [26]. In addition, the -857T (but not the -857C) allele strongly binds the transcription factor OCT1, which blocks the interaction of nuclear factor-kappa-B (NF-κB) to the nearby region -873 to -863, thereby inhibiting *TNF-α *transcription [27]. To conclude, the data gathered so far favor a possible influence of the -308 and the -857 *TNF-α *promoter positions on the production of TNF-α, consistent with the association that we found with RA long-term outcome measures.

Another nearby SNP, at position -863, is involved in NF-κB binding, and a report has suggested that the rare -863A allele is associated with a lower transcriptional activity [27], which can be inferred as an indication of possibly higher disease activity and worse prognosis in patients with the -863CC genotype. This interpretation is consistent with the association trend that we have depicted between this genotype and RF, a well-known RA prognostic factor.

One of the most studied *TNF-α *gene polymorphisms is the one in position -238 (G→A). Different authors have associated both the -238A and -238G allelic forms to high TNF-α production but with clearly contrasting results [28,29]. A significant number of variables may contribute to this apparent contradiction, including differences in cell line types, the length of the promoter sequence, and the presence/absence of the 3' untranslated region [30]. Regardless of whether there is a direct functional effect of this SNP, some studies have shown an association of the -238GG genotype with worse RA prognosis [31,32], so the trend that our study has shown for an association of this genotype with HAQ score and RF is not surprising.

## Conclusion

Although genetic influences on RA outcome remain incompletely understood, our results suggest that *TNF-α *gene promoter polymorphisms influence the outcome of this chronic disease. Despite this evidence, the value of genotyping RA patients in order to define their clinical course will remain unproven until a proper prospective evaluation of this cohort of patients validates this hypothesis.

## Abbreviations

ACR = American College of Rheumatology; DAS28 = disease activity score using 28 joint counts; DD = disease duration; DMARD = disease-modifying anti-rheumatic drug; EM = expectation-maximization; ESR = erythrocyte sedimentation rate; HAQ = Health Assessment Questionnaire; NF-κB = nuclear factor-kappa-B; PCR = polymerase chain reaction; RA = rheumatoid arthritis; RF = rheumatoid factor; SNP = single-nucleotide polymorphism; SvdH = Sharp/van der Heijde; TNF-α = tumor necrosis factor-alpha.

## Competing interests

The authors declare that they have no competing interests.

## Authors' contributions

JEF conceived the design of the study, coordinated all of the laboratorial work, personally observed most of the patients and coordinated and trained others to perform the same observations, coordinated and trained observers for the use of the SvdH method, participated in the statistical analysis, and coordinated all phases of manuscript writing. JC and JT carried out the molecular genetic studies, participated in the statistical analysis, and helped to draft the manuscript. ES carried out the x-ray evaluation using the SvdH method. VLA, MA, and MAA-T participated in the statistical analysis and helped to draft the manuscript. HC participated in the clinical data collection and helped to draft the manuscript. AFM, MS, PN, MJS, AM, MC, RM, A Braña, LM, JVP, A Barcelos, JCdS, LMS, GF, MR, HJ, and AQ participated in the clinical data collection. JL participated in the x-ray evaluation using the SvdH method. JC-L and PW participated in the molecular genetic studies. TC coordinated and designed part of the laboratorial work. JAPdS, JB, and MVQ participated in the design of the study and in the draft of the manuscript. All authors read and approved the final manuscript.
